# Protocol for ultracentrifugation-based isolation and characterization of extracellular vesicles from FGF2-primed astrocytes

**DOI:** 10.1016/j.xpro.2026.104733

**Published:** 2026-07-24

**Authors:** Xiaomin Wen, Mingming Wang, Zihao Gao, Wanjun Cao, Shengxi Wu, Ye Xi

**Affiliations:** 1 Department of Neurobiology, School of Basic Medicine, Fourth Military Medical University, Xi’an, Shaanxi 710032, P.R. China; 2 The Shaanxi Province Key Laboratory of Brain Function Analysis and Modulation, Xi’an, Shaanxi 710032, P.R. China

**Keywords:** Cell culture, Cell isolation, Cell separation/fractionation, Neuroscience

## Abstract

Extracellular vesicles (EVs) mediate intercellular communication by transferring bioactive molecular cargo. We present a protocol for isolating EVs from fibroblast growth factor-2 (FGF2)-primed primary mouse astrocytes using differential ultracentrifugation. We describe steps for primary astrocyte culture, FGF2 priming, EV isolation, and comprehensive characterization encompassing transmission electron microscopy (TEM). We then detail procedures for nanoparticle tracking analysis (NTA), western blotting, and mass spectrometry-based proteomic analysis. EVs isolated by this approach protect against mitochondrial and synaptic toxicity in Parkinson’s disease models.

For complete details on the use and execution of this protocol, please refer to Wen et al.^1^

## Highlights

• Protocol for extracting and culturing primary astrocytes from neonatal mouse cortex
• EV isolation from FGF2-primed astrocytes by differential ultracentrifugation
• EV characterization by TEM, NTA, and western blotting
• Proteomic analysis of EVs using Orbitrap Fusion Lumos Mass spectrometer

## Abstract (editor highlights)

Publisher’s note: Undertaking any experimental protocol requires adherence to local institutional guidelines for laboratory safety and ethics.

## Graphical abstract

**Figure:**
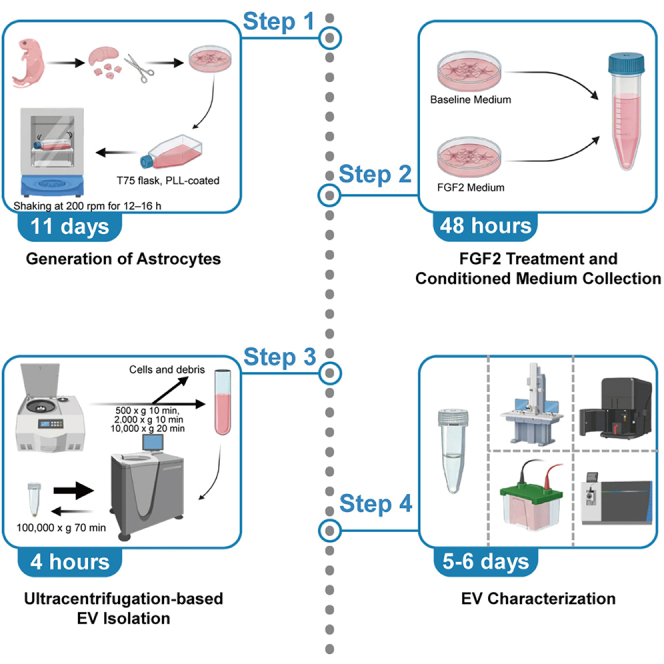

## Before you begin

Astrocyte-derived extracellular vehicles (EVs) are important mediators of astrocyte-to-neuron communication and can modulate the neuronal microenvironment under physiological and pathological conditions. EVs shuttle various proteins and nucleic acids between cells, playing crucial roles in maintaining cellular homeostasis, mediating immune responses, and facilitating cell-to-cell communication in the central nervous system. In Parkinson’s disease (PD), mitochondrial morphological and functional damage represents a key pathogenic mechanism leading to dopaminergic neuronal degeneration.^2^^,^^3^ Consequently, EV-based therapeutic strategies that can protect mitochondrial integrity in dopaminergic neurons are of significant interest.^4^ In neurodegenerative diseases such as PD, astrocytes often develop reactive phenotypes that contribute to neuronal toxicity.^5^^,^^6^ We recently demonstrated that priming astrocytes with fibroblast growth factor-2 (FGF2), a key regulator of astrocytic differentiation, induces these cells to develop neuroprotective phenotypes and release EVs with distinct cargo profiles. FGF2-primed astrocyte-derived EVs (FGF2-EVs) exhibit superior beneficial effects compared to EVs from naïve astrocytes, protecting dopaminergic neurons against mitochondrial fragmentation and stimulating synaptogenesis in PD models. These findings indicate that FGF2-EVs could serve as an optimized therapeutic approach for PD.

In this protocol, we describe the isolation of EVs from FGF2-primed primary mouse astrocytes by differential ultracentrifugation, followed by EV characterization using transmission electron microscopy (TEM), nanoparticle tracking analysis (NTA) and Western blotting to assess EV identity, purity, and morphology, as well as mass spectrometry-based proteomic analysis to define cargo protein profiles and identify differentially enriched proteins that may underlie the distinct neuroprotective effects of FGF2-EVs. Although this protocol was established for FGF2-primed primary astrocytes, the workflow can be adapted for EV isolation and characterization from other glial cell types. In addition, the procedures for primary astrocyte generation and culture described here may be useful for investigating astrocyte function in other neurodegenerative disease contexts.

Nomenclature: Current characterization methods cannot specifically distinguish the subcellular origin of vesicles isolated by ultracentrifugation. In accordance with the International Society for Extracellular Vesicles (ISEV) guidelines, we collectively refer to this material as EVs.^7^

### Innovation

This protocol introduces FGF2 priming as a key modification to standard astrocyte-derived EV isolation workflows. While conventional protocols collect EVs from naïve astrocytes, preconditioning primary mouse astrocytes with FGF2—a key regulator of astrocytic differentiation—reprograms these cells toward a neuroprotective phenotype, thereby yielding FGF2-EVs with enhanced neuroprotective capacity against mitochondrial fragmentation and improved capacity to stimulate synaptogenesis.

The protocol provides detailed procedures for primary astrocyte isolation from neonatal mouse cortex, FGF2 priming, and EV isolation by differential ultracentrifugation. The FGF2-priming step is simple, reproducible, and requires minimal additional time within the isolation workflow.

For comprehensive characterization, we integrate transmission electron microscopy (TEM), nanoparticle tracking analysis (NTA), and Western blotting to assess EV identity, purity, and morphology, together with mechanistic investigation of how astrocyte phenotypic modulation alters EV cargo composition to identify specific bioactive molecules responsible for neuroprotection.

Although established for FGF2-primed primary astrocytes, this optimized workflow can be readily adapted for EV isolation from other primed or polarized glial cell types, offering a flexible platform for generating cell-specific EV preparations with defined functional cargo.

### Institutional permissions

Animal experiments were conducted in accordance with the guidelines of the Institutional Animal Care and Use Committee at the Fourth Military Medical University and adhered to the Animal Research Reporting of In Vivo Experiments Guidelines (FMMULL-20220930). All animals were housed in the Specific Pathogen Free (SPF) Animal Center of the Fourth Military Medical University.

### Preparation of extracellular vesicle-depleted fetal bovine serum

**Timing: 1 day**

***Note:*** To save time and cost, use commercially available extracellular vesicle-free serum.

1. Thaw the cell culture serum naturally at 4°C, and mix thoroughly before centrifugation.
2. Remove precipitates by centrifugation at 2,000 × *g* for 10 min at 4°C.
3. Further remove residual precipitates by centrifugation at 10,000 × *g* for 30 min at 4°C.
4. Ultracentrifuge at 100,000 × *g* for 12 h at 4°C using a Type 45 Ti fixed-angle rotor in sterile #345776 centrifuge tubes (sterile, enzyme-free; #342697 adapters are required) to complete serum purification.^8^

***Note:*** If sterile tubes are unavailable, use autoclave-compatible centrifuge tubes and sterilize them by autoclaving.

5. Collect the supernatant in a biosafety cabinet and store at −20°C before use.

***Note:*** Use the appropriate rotor and tubes as specified and balance them; perform liquid transfer in a biosafety cabinet to ensure effective removal of contaminants and maintain sterility. To avoid repeated freeze-thaw cycles, aliquot the supernatant into single-use volumes (e.g., 0.5 mL or 1 mL) in sterile tubes prior to freezing. Label each aliquot with volume, date, and sample information.

### Preparation of reagents, tools, and equipment

**Timing: 2 days**

6. Sterilize surgical scissors and forceps by autoclaving. Use sterile, pre-packaged microcentrifuge tubes.
7. Prewarm astrocyte medium and sterile PBS in a 37°C water bath before starting the experiment.
8. Pre-cool centrifuges and their rotors to 4°C.

**Figure 1:**
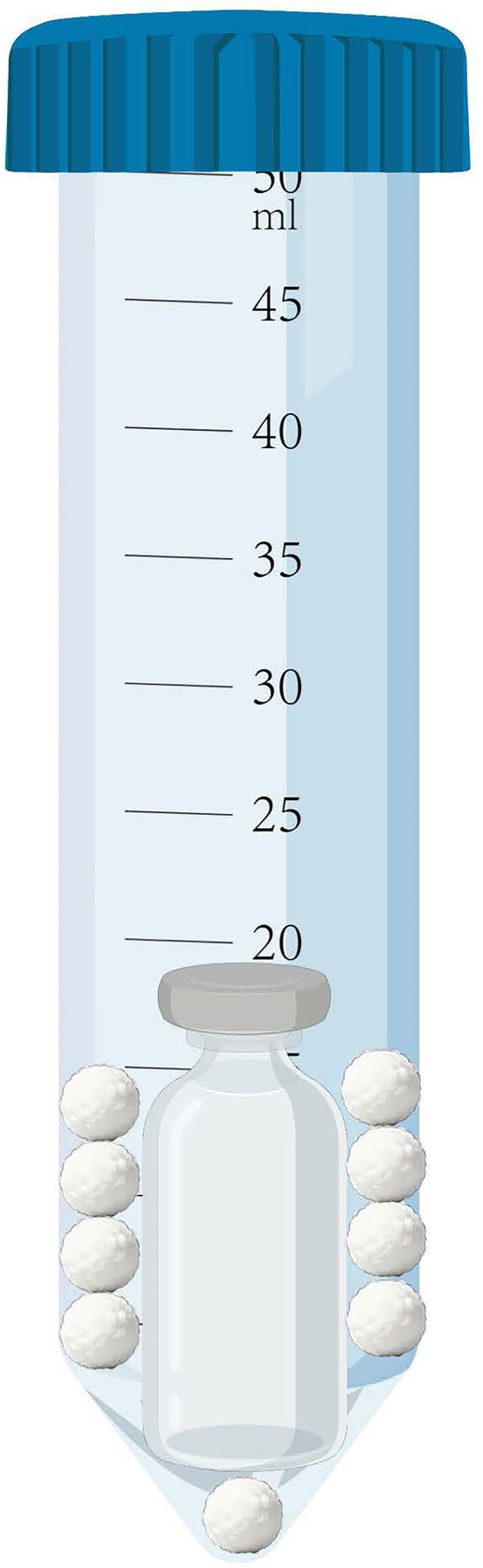
Illustration of loading centrifuge
9. Dissolve fibroblast growth factor-2 (FGF2).
  a. Remove the ampoule containing FGF2 from 4°C, place it into a 50 mL centrifuge tube, and centrifuge at 3,000 × *g* (such as HERMLE Z 32 HK) for 10 min at 4°C.
    ***Note:*** Fill the centrifuge tube with cotton or foam to cushion the ampoule (Figure 1).
  b. Dissolve the powder in sterile, nuclease-free water to a stock concentration of 200 μg/mL (or as recommended by the manufacturer), and mix by pipetting until the powder is completely dissolved.
    ***Note:*** Gently open the rubber stopper to prevent powder spillage.
  c. Securely close the rubber stopper, place the ampoule back into a 50 mL centrifuge tube, and centrifuge at 3,000 × *g* (such as HERMLE Z 32 HK) for 10 min at 4°C.
  d. Aliquot the solution into sterile centrifuge tubes and store at −80°C for long-term preservation.
    ***Note:*** Avoid repeated freeze-thaw cycles to maintain protein activity. Use screw-cap tubes (e.g., 0.5 mL) with low protein-binding surface to prevent protein loss and contamination. Refer to the manufacturer's instructions for detailed reconstitution steps.
10. Coat T75 culture flask.
  a. Prepare borate buffer.
    i. Dissolve 0.68 g boric acid and 0.86 g sodium tetraborate decahydrate in 100 mL of sterile triple-distilled water.
    ii. Filter the buffer through a 0.22 μm filter before use to ensure sterility and remove particulates, then store at −20°C.
  b. Dissolve 5 mg of poly-L-lysine (PLL) in 50 mL of boric acid buffer to obtain a 4 × PLL solution.
  c. Dilute PLL to 1 × with sterile triple-distilled water.
  d. Add the diluted PLL (at least 5 mL) to a T75 flask to completely cover the bottom surface, and incubate at 37°C for 12–16 h.
    ***Note:*** Ensure the entire bottom surface is evenly covered for uniform coating.
  e. After incubation, rinse the flask three times with sterile water (at least 5 mL).
  f. Air-dry the flask in a biosafety cabinet with the lid removed; place the lid inside the cabinet as well.
    ***Note:*** Seal the flask opening tightly with parafilm after coating and store at 4°C.

## Key resources table

**Table:** 

REAGENT or RESOURCE	SOURCE	IDENTIFIER
**Antibodies**		

anti - CD81(1:5000)	Proteintech	Cat No: 27855-1-AP; RRID: AB_2880995
anti - CD9(1:5000)	Proteintech	Cat No: 20597-1-AP; RRID: AB_2878706
anti - TSG101(1:5000)	Proteintech	Cat No: 67381-1-Ig; RRID: AB_2882628
anti - Calnexin(1:5000)	Proteintech	Cat No: 10427-2-AP; RRID: AB_2069033
anti - GFAP(1:200)	Cell signaling Technology	Cat No: 80788T; RRID: AB_2799963

**Chemicals, peptides, and recombinant proteins**		

FGF2	Sino Biological	10014-HNAE
Dulbecco’s Modified Eagle’s Medium/Nutrient Mixture F-12 Ham	Sigma	D8437
Poly-L-lysine	Sigma-Aldrich	P6282-5MG
Pen/Strep glutamine (100 ×)	Gibco	10378-016
Fetal bovine serum	Gibco	10099141C
L-Glutamine 200 mM (100 ×)	Gibco	25030-081
Boric acid	Sigma	B6768
Sodium tetraborate decahydrate	Sigma	B9876
1 × PBS	Gibco	10010023
1 × HBSS	Gibco	14175095
0.25% Trypsin-EDTA (1 ×)	Gibco	25200-056
TBST	Biosharp	BL1683A
WesternLumaxLight Superior HRP substrate	Zeta-Life	310208
Skim milk powder	Wondersun	N/A
SDS-PAGE sample loading buffer, 5 ×	Epizyme Biotech	LT101

**Critical commercial assays**		

Pierce BCA protein assay kit	Thermo Fisher Scientific	23225

**Experimental models: Organisms/strains**		

Mouse: C57BL/6 wild-type, 8-10 weeks old, males and females	SPF Animal Center of the Fourth Military Medical University	N/A

**Other**		

0.22 μm sterile syringe filter	Millipore	SLGPR33RB
Nunc™ EasYFlask™ 75 cm^2^	Thermo Fisher Scientific	156499
Nunc™ EasYDish™ culture dish	Thermo Fisher Scientific	150462
50 mL centrifuge tube	Corning	430829
Centrifuge	HERMLE	Z 32 HK
Ultracentrifuge	BECKMAN	OPTIMA XPN-100
Type 45 Ti fixed-angle rotor	BECKMAN	339160
Sterile and certified-Free tubes	BECKMAN	345776
Type 70 Ti fixed-angle rotor	BECKMAN	337922
Thickwall polycarbonate tubes	BECKMAN	355631
Transmission electron microscopy	FEI	Tecnai G2 Spirit BioTwin
Nanoparticle tracking analysis	Particle Metrix	ZetaVIEW S/N 17-310
PVDF membrane	Millipore	IPFL00010
Chemiluminescent imaging device	Bio Rad	ChemiDocTM XRS+
Mass spectrometer	Thermo Fisher Scientific	Orbitrap Fusion Lumos
3 kDa ultrafiltration spin column	Thermo Fisher Scientific	88512
iST Sample Preparation kit	PreOmics	P.O.00027
Pierce™ Quantitative Colorimetric Peptide Assay	Thermo Fisher Scientific	23275

## Materials and equipment

Prepare the astrocyte culture medium by combining the following components under sterile conditions:

**Table:** Astrocyte medium

Reagent	Final concentration	Amount
Dulbecco’s Modified Eagle’s Medium/Nutrient Mixture F-12 Ham	N/A	88 mL
Fetal Bovine Serum	10%	10 mL
Penicillin-Streptomycin	1%	1 mL
L-Glutamine	1%	1 mL
**Total**	N/A	**100 mL**

Store at 4°C for up to 14 days.

**CRITICAL:** Mix all components of the astrocyte culture medium thoroughly. For EV collection, prepare the culture medium using extracellular vesicle-free serum instead of regular serum.

## Step-by-step method details

### Generation of primary mouse astrocytes

**Timing: 11 days**

**Figure 2:**
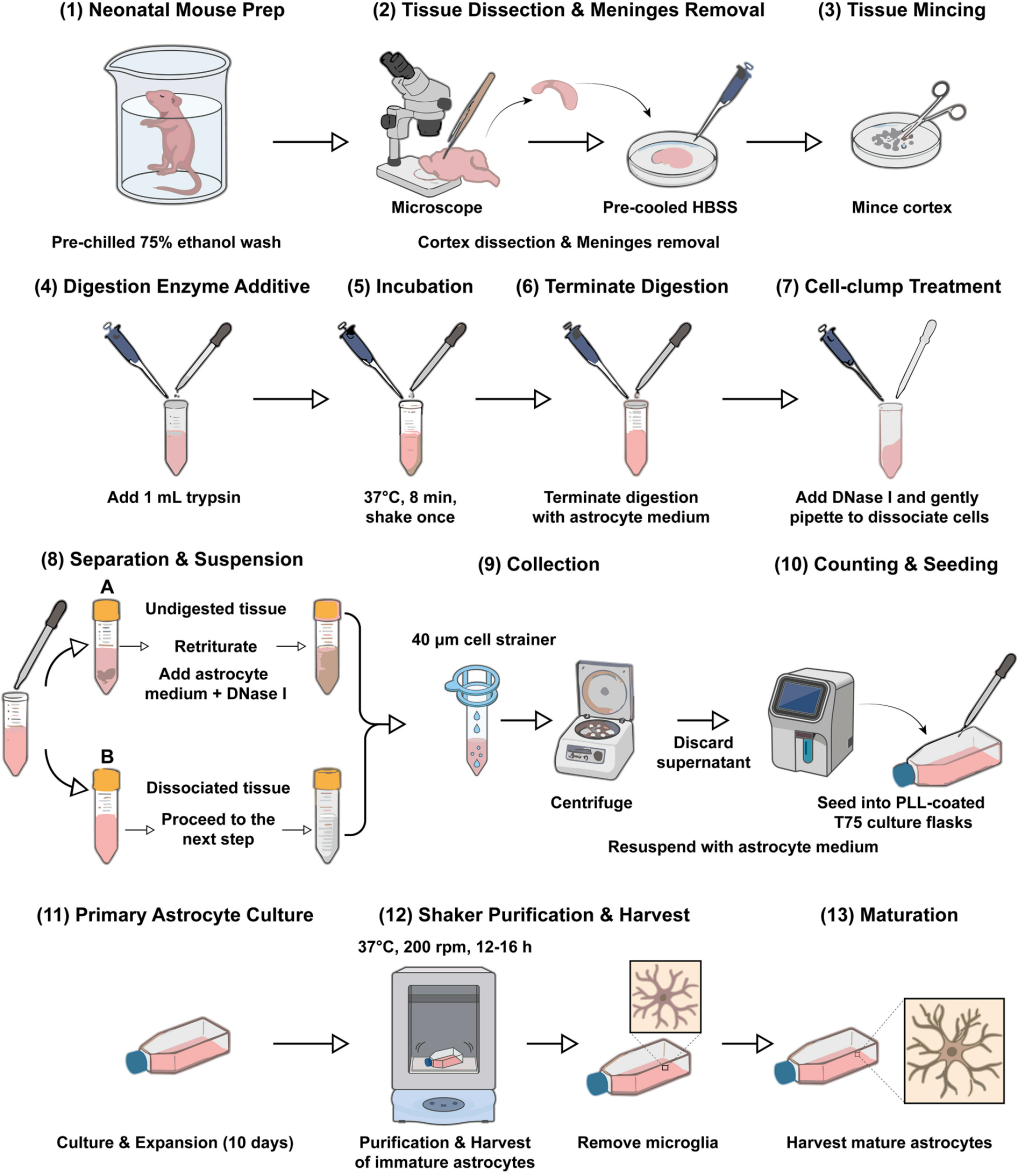
Generation of primary mouse astrocytes Illustrative overview of astrocyte generation.

This step describes the isolation of cerebral cortical tissue from neonatal mice and the establishment of primary astrocyte cultures (Figure 2).

1. Anesthetize the neonatal C57BL/6 mice (postnatal day 0–3, P0-P3) with 1.5% isoflurane delivered using a gas anesthesia machine at an oxygen flow rate of 1.0 L/min until spontaneous movement ceases.

***Note:*** Monitor the mouse status carefully and wait approximately 5 min for the anesthetic to take effect.

2. Rinse the mice with pre-cooled (4°C) 75% ethanol.

***Note:*** Disinfect the mice skin thoroughly. Spray or gently wipe the entire skin surface with ethanol-soaked gauze, repeating at least twice.

3. Dissect to expose the brain.

***Note:*** Perform the dissection under a stereomicroscope and on an ice bed, and periodically recheck reflexes during dissection.

4. Carefully remove the meninges, isolate the cortical tissue using fine surgical instruments and transfer the tissues into a 6 cm dish containing ice-cold HBSS.

**Figure 3:**
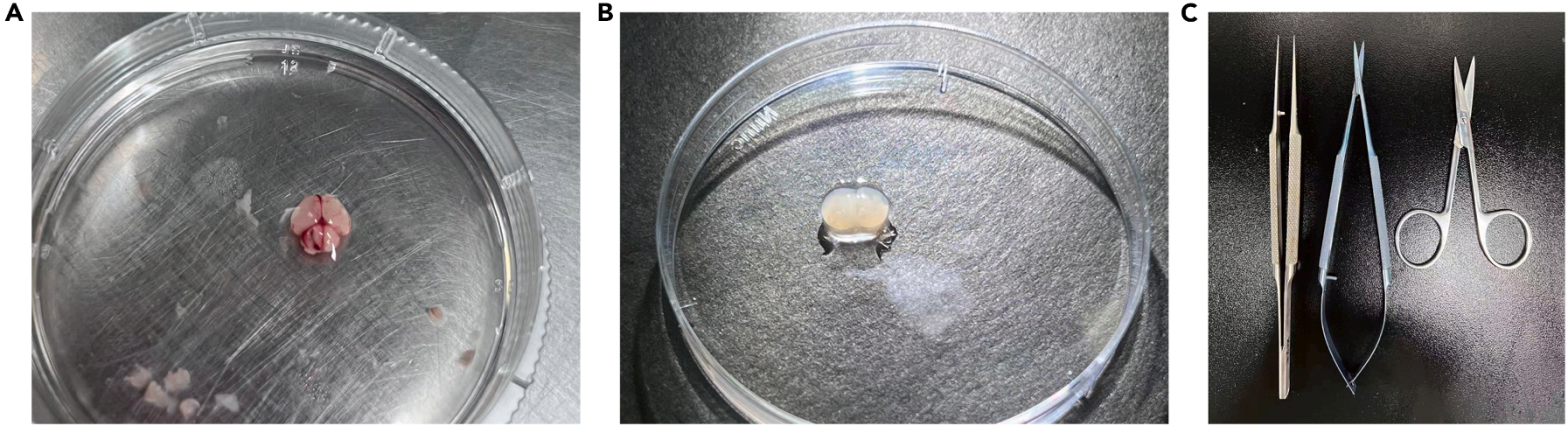
Isolation of mouse cortical tissue (A) Isolate the brain tissue from neonatal mice. (B) Carefully isolate the cortical brain tissue using ophthalmic forceps and remove the meninges. (C) The instruments used in this step.

***Note:*** Carefully remove the meninges to avoid fibroblast contamination (Figure 3). Maintain the dish on ice for all subsequent steps.

5. Mince the cortical tissues into small pieces (approximately 1–2 mm^3^) using micro-dissecting scissors.

***Note:*** Discard excess HBSS, leaving approximately 500 μL to cover the tissue.

6. Add 1 mL 0.25% trypsin and digest the tissues at 37°C for 8 min. At the 4-min time point, remove the dish and gently shake it by hand 3–5 times to agitate the tissues.
7. Stop digestion by adding 2 mL astrocyte medium.
8. Gently pipette the tissue solution up and down using a glass Pasteur pipette until the tissue dissociates into a single-cell suspension. Transfer the supernatant to a 15 mL centrifuge tube.

***Note:*** Add DNase I (1:1000) to reduce cell aggregation.

9. Add fresh medium and DNase I (1:1000) to the remaining tissue and gently pipette again until a uniform cell suspension is obtained, with no visible tissue fragments remaining.
10. Filter the cell suspension through a 40 μm cell strainer and centrifuge the filtrate at 900 × *g* for 5 min.
11. Discard the supernatant and resuspend the cells using astrocyte medium.
12. Transfer the cell suspension from Step 11 to PLL-coated T75 flasks and adjust the total volume to 25 mL with astrocyte medium.

***Note:*** Seed cells isolated from cortical tissues of 3–4 pups into one T75 culture flask.

13. Culture the primary astrocytes in astrocyte medium for 10 days.

***Note:*** Replace half of the astrocyte medium every 3 days.

14. Shake the flasks at 37°C for 12–16 h on a constant-temperature shaker at 200 rpm to remove microglia and oligodendrocytes. Replace the culture medium after shaking to obtain purified immature astrocytes.

**CRITICAL:** Passage primary cultured astrocytes to the third generation before use. For all experiments, use astrocytes within five passages.

### Treatment of primary mouse astrocytes with FGF2

**Timing: 2 days**

Fibroblast growth factor-2 (FGF2), a key factor for astrocyte development, reverses the toxic phenotype of reactive astrocytes. This section describes FGF2 treatment of primary mouse astrocytes.

15. Remove the astrocyte medium and wash the cells three times with PBS.

**CRITICAL:** Thorough washing is essential to remove residual EVs from serum-containing medium, ensuring accurate assessment of cell-derived EV effects.

16. Add DMEM/F-12 containing 10% extracellular vesicle-free serum and L-Glutamine.

***Note:*** No Pen-strep were added to the medium during EV harvest, so as to exclude potential interference.

17. For FGF2-EV isolation, supplement the medium with FGF2 to a final concentration of 2 ng/mL.
18. Incubate the cells for 48 h and then collect the conditioned medium in a biosafety cabinet into a 50 mL centrifuge tube.
  a. Centrifuge the collected medium at 300 × *g* for 10 min at 4°C to pellet dead cells and large debris.
  b. Transfer the supernatant to a new sterile centrifuge tube.
  c. If EV isolation cannot be completed on the same day, store the clarified supernatant at −80°C.

***Note:*** Use freshly collected conditioned medium for EV preparation. Before the isolation procedure, thaw samples thoroughly at 4°C to preserve vesicle integrity and minimize degradation.

### FGF2-EV preparation

**Timing: 4–6 h**

This step describes the isolation of FGF2-EVs from conditioned medium by sequential centrifugation and ultracentrifugation. The purified EVs are then resuspended in sterile PBS for downstream analysis.

19. Pre-cool all centrifuges to 4°C before use.
20. Centrifuge the collected medium at 500 × *g* for 10 min.

**CRITICAL:** This step aims to remove the dead cells.

21. Transfer the supernatant to a new 50 mL centrifuge tube and centrifuge at 2,000 × *g* for 10 min.

**CRITICAL:** This step aims to remove cell debris and organelles.

22. Transfer the supernatant to a new 50 mL centrifuge tube and centrifuge at 10,000 × *g* for 20 min at 4°C.

**CRITICAL:** This step aims to remove cell debris and cell membrane components.

23. Transfer the supernatant to ultracentrifuge tubes (#355631) and ultracentrifuge at 100,000 × *g* for 70 min at 4°C using a Type 70 Ti fixed-angle rotor.

***Note:*** Balance tube weights using an analytical balance. If liquid volumes are unequal, adjust with PBS to balance precisely and prevent equipment damage.

24. Discard the supernatant, resuspend the pellet in PBS (appropriate volume, e.g., 20 mL), and ultracentrifuge again at 100,000 × *g* for 70 min at 4°C.

***Note:*** Use gentle pipetting to mix the sample and avoid vortexing to prevent vesicle damage or aggregation.

25. Discard the supernatant and resuspend the final pellet in 100 μL sterile PBS for subsequent experiments.
26. Determine the concentration of isolated EVs using a BCA protein assay kit.

***Note:*** EVs derived from two T75 flasks typically exhibit a particle concentration above 1 × 10^10^ particles/mL, with a measured protein concentration of approximately 1 mg/mL.

27. Prepare 20 μL of the resuspended EV sample for TEM and Western blotting, and 10 μL for NTA.

***Note:*** Use freshly prepared samples for optimal sample quality and reliable results.

28. Aliquot the isolated EVs and store at −80°C for subsequent experiments.

***Note:*** Avoid repeated freeze-thaw cycles. Samples can be stored for up to 3 months.

29. Dilute FGF2-EVs in an appropriate solution and filter through a 0.22 μm filter before use.

**CRITICAL:** When the liquid volume is extremely small (e.g., <100 μL), filtration may result in substantial loss of EVs. In such cases, it is recommended to dilute the EVs in the culture medium before conducting the filtration process.

### Characterization of EVs by TEM

**Timing: 5–6 h**

This step describes the characterization of FGF2-EVs using transmission electron microscopy (TEM).

**Figure 4:**
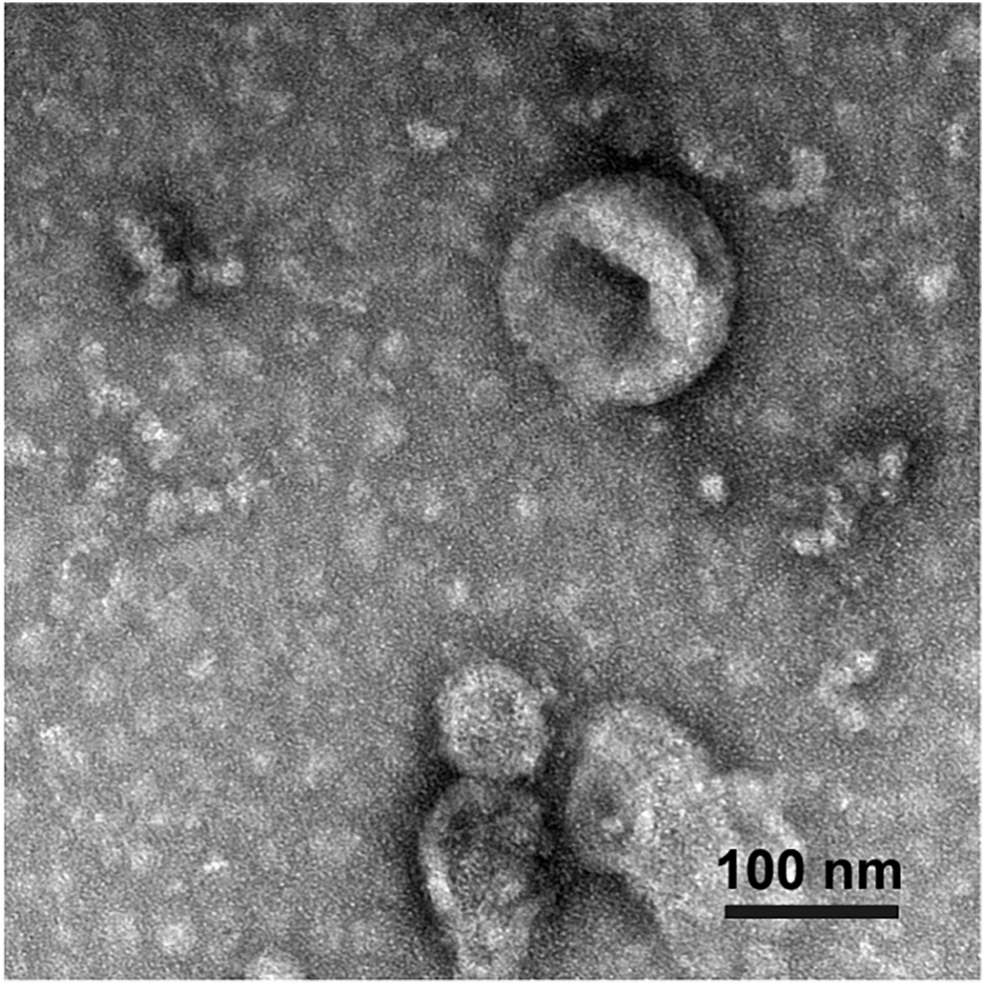
TEM image of FGF2-EVs EVs isolated from FGF2-primed astrocytes were imaged by TEM. Scale bar: 100 nm.

30. Thaw the frozen EV sample in a 25°C water bath, then transfer it to an ice box to maintain a low temperature.
31. Place 5 μL of the EV sample onto a copper grid and allow adsorption for 5 min at 22°C–25°C.
32. Remove unbound liquid by gently touching the edge of the grid with filter paper.
33. Add 2% uranyl acetate solution and incubate for 1 min.
34. Remove unbound stain using filter paper.
35. Allow the grid to air-dry for 20 min at 22°C–25°C before observation and image acquisition under TEM operated at an accelerating voltage of 80 kV. Acquire images at magnifications of 30,000×–50,000× to visualize typical cup-shaped or round membranous vesicle structures (Figure 4).

**CRITICAL:** Avoid introducing air bubbles during liquid application, as this may interfere with sample adsorption and staining uniformity.

### Identification of EVs by NTA

**Timing: ∼30 min/sample**

This step describes the characterization of FGF2-EVs using nanoparticle tracking analysis (NTA).

**Figure 5:**
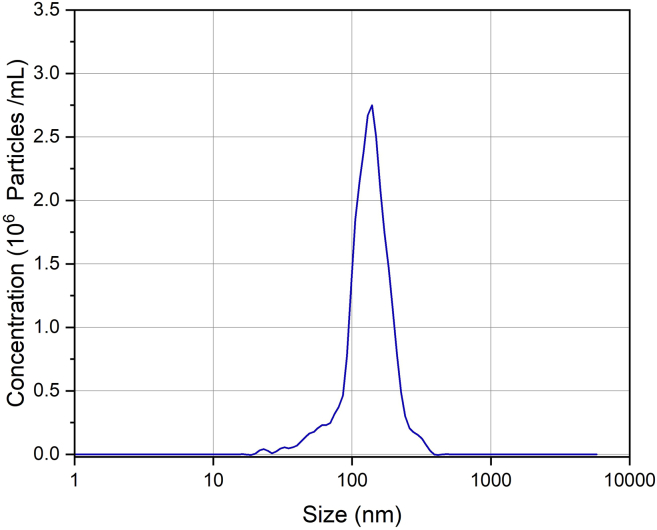
NTA result of FGF2-EVs Particle size distribution of EVs isolated from FGF2-primed astrocytes, measured by NTA. The result shows a unimodal size distribution with a major peak at ∼130 nm.

36. Thaw frozen EV samples in a 25°C water bath and immediately place on ice to minimize aggregation and degradation.
  a. Dilute the EV suspension with sterile, 0.22 μm-filtered 1× PBS.
  b. As a starting point, dilute approximately 1,000-fold (typical range: 500- to 2,000-fold) to reach a final particle concentration of 1×10^7^–1×10^8^ particles/mL, which is the optimal range for the instrument.
37. Perform NTA measurement using the ZetaVIEW S/N 17-310 instrument and analyze data using ZetaView 8.04.02 software (Figure 5).

**CRITICAL:** Load the diluted sample slowly into the measurement cell to avoid bubble formation. For each sample, measure at least three independent aliquots to ensure reproducibility.

***Note:*** Optimize the dilution factor based on particle concentration to ensure accurate tracking and reliable results.

### Identification of EVs by western blotting

**Timing: 2 days**

This step describes the characterization of FGF2-EVs using Western blotting.

38. Keep the EV sample on ice. Mix an aliquot containing 20 μg of total EV protein (or approximately 8 × 10^8^ particles) with sodium dodecyl sulfate-polyacrylamide gel electrophoresis (SDS-PAGE) sample loading buffer according to a final 1× concentration, and heat at 97°C for 5 min.

***Note:*** The recommended protein amount (20 μg) serves as a starting point for standard SDS-PAGE and Western blotting; the optimal amount may need empirical adjustment depending on the abundance of the target protein. Maintain the EV sample on ice at all times before heating to prevent protein degradation.

**Figure 6:**
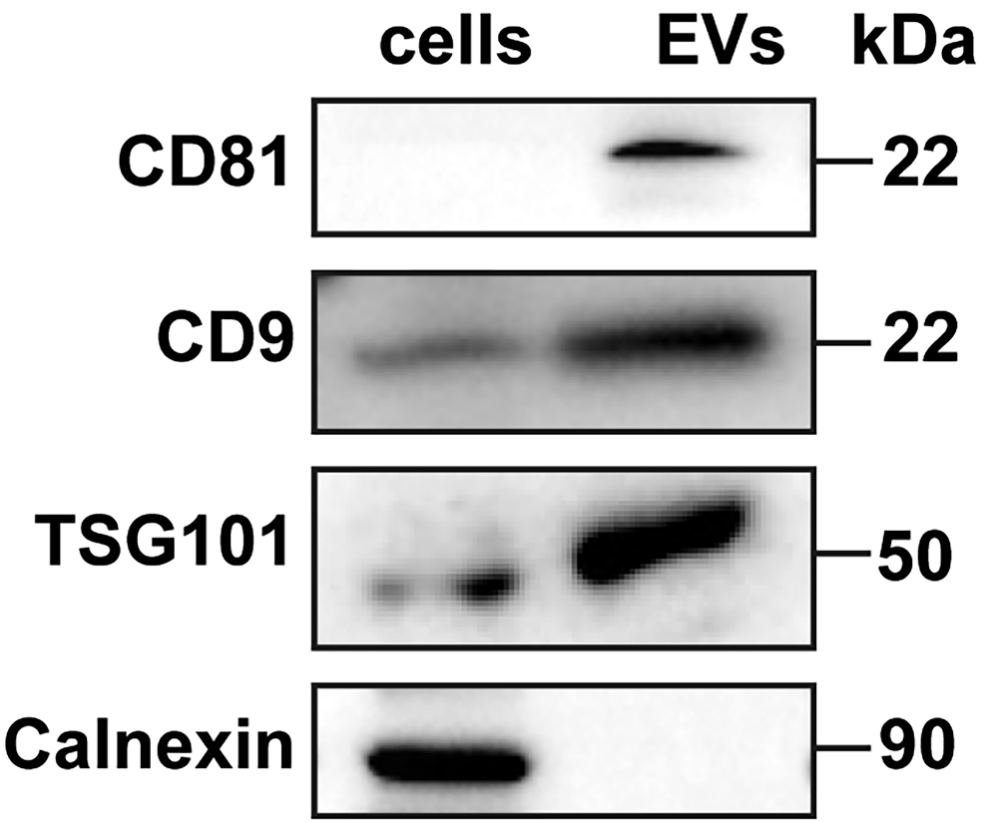
Western blotting analysis of FGF2-EV markers Western blotting analysis of CD81, CD9, TSG101 and Calnexin in control cell lysates and FGF2-EV samples.

39. Separate sample proteins by SDS-PAGE using a Tris-glycine-SDS running buffer (25 mM Tris, 192 mM glycine, 0.1% SDS, pH 8.3) at 80 V (stacking) and then 120–130 V (resolving).
40. Activate the polyvinylidene fluoride (PVDF) membrane in methanol for 30 s, rinse, and equilibrate in transfer buffer (25 mM Tris, 192 mM glycine, 20% methanol, pH 8.3). Electroblot at 300 mA for 120–180 min in an ice-water bath.
41. Block the membrane with 5% skim milk powder in TBST (0.1% Tween-20) for 2 h at 22°C–25°C.
42. Incubate the membrane at 4°C for 12–16 h with primary antibodies: anti-CD81 (1:3000 dilution), anti-CD9 (1:3000 dilution), anti-TSG101 (1:3000 dilution), and anti-Calnexin (1:5000 dilution) diluted in TBST.
43. Wash the membrane three times with TBST, 10 min each time.
44. Incubate with horseradish peroxidase-conjugated goat anti-mouse/rabbit IgG secondary antibodies (1:5000 dilution) for 2 h at 22°C–25°C.
45. Detect signals using the ChemiDoc™ XRS+ system with WesternLumaxLight Superior HRP substrate (Figure 6).

### Proteomic analysis of EVs by LC-MS/MS

**Timing: 2–3 days**

This step describes the proteomic workflow used to identify differentially expressed proteins in EVs from naïve and FGF2-primed astrocytes. Peptides are fractionated and analyzed by LC-MS/MS, followed by DDA data processing and functional enrichment analysis.

46. Lyse EVs in lysis buffer containing 1% sodium deoxycholate, 8 M urea and 1M TEAB for sample preparation.
  a. Use an EV amount equivalent to 50–100 μg of total protein as the starting material, and vortex until complete dissolution.
  b. Lyse samples at 4°C for 30 min, centrifuge at 12,000 × *g* for 20 min at 4°C, and concentrate the supernatant using a 3 kDa ultrafiltration spin column.
  c. Determine protein concentration using a BCA Protein Assay Kit.
47. Prepare peptides using an iST Sample Preparation kit.
  a. Add 50 μL lysis buffer, shake at 1000 rpm for 10 min at 95°C, then cool to 22°C–25°C.
  b. Add trypsin at an enzyme-to-substrate ratio of 1:50 and incubate at 500 rpm for 2 h at 37°C; stop digestion with stop buffer.
  c. Desalt on the iST cartridge, elute with 2 × 100 μL elution buffer.
48. Quantify peptides using the Pierce™ Quantitative Colorimetric Peptide Assay, then dry the peptide samples in a vacuum concentrator. Store the dried peptides at −80°C until further use.
49. Fractionate the resulting peptides using high-pH reversed-phase chromatography.
  a. Resuspend in 20 mM ammonium formate, pH 10.0.
  b. Separate on the Ultimate 3000 system connected to XBridge C18 column (4.6 mm × 250 mm, 5 μm).
50. Analyze the fractionated peptides by LC-MS/MS on an Orbitrap Fusion Lumos mass spectrometer coupled to an EASY-nLC 1200 system.
  a. Resuspend the dried peptides in 0.1% formic acid aqueous solution and inject onto an Acclaim PepMap C18 analytical column (75 μm × 25 cm).
  b. Set DDA parameters: MS (m/z 350–1500, resolution 120,000, automatic gain control (AGC) 4 × 10^5^, max injection 50 ms, dynamic exclusion 30 s); HCD-MS/MS (resolution 15,000, AGC 5 × 10^4^, max injection 35 ms, collision energy 32).
51. Process the DDA data in Spectronaut X software, searching the UniProt database.
  a. Set carbamidomethyl (C, +57.02 Da) as the fixed modification.
  b. Set oxidation (M, +15.99 Da) as the variable modification.
  c. Filter peptides by 1% FDR and filter proteins by 1 unique peptide.
52. Select proteins significantly upregulated in FGF2-EVs for downstream GO and KEGG enrichment analysis.

## Expected outcomes

**Figure 7:**
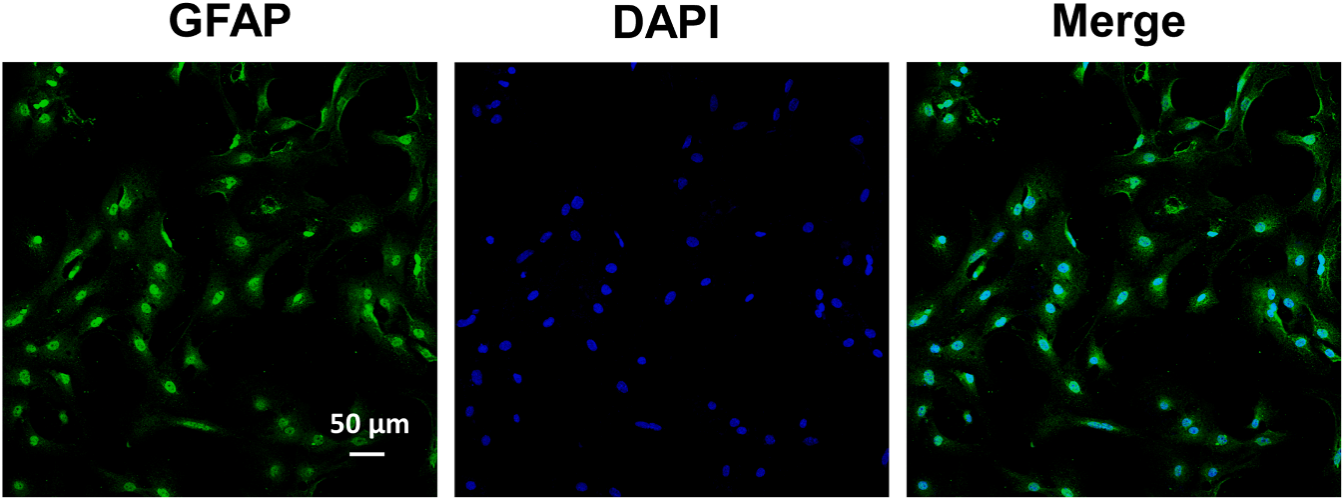
Identification of astrocytes Immunostaining of GFAP in primary astrocytes. Scale bar: 50 nm.

Using this protocol, researchers can isolate primary astrocytes from mouse cortex, perform FGF2 priming and subsequent isolation of astrocyte-derived extracellular vesicles (FGF2-EVs). To ensure priming reproducibility, FGF2 priming was performed for at least 24 h in cultures displaying normal morphology/viability with no obvious cell detachment, and with clear culture medium before EV collection. The feasibility of primary astrocyte isolation is shown in Figure 7. Isolated FGF2-EVs exhibit characteristic features of small extracellular vesicles: TEM reveals the typical morphology of purified FGF2-EVs (Figure 4), NTA determines their size distribution and particle concentration (Figure 5), and Western blotting confirms the presence of EV-associated markers, including CD81, CD9, and TSG101 (Figure 6). These results indicate successful isolation and characterization of FGF2-EVs. Although this protocol was developed for astrocyte-derived EVs, the overall workflow may also be adaptable to EVs studies in other cell types.

## Limitations

This protocol relies on ultracentrifugation for EV isolation, which, while widely used, co-precipitates vesicles of varying sizes and may co-isolate non-vesicular components such as protein aggregates or lipoproteins, potentially affecting downstream analyses. Additionally, primary astrocyte cultures exhibit inherent biological variability between isolations, which may influence EV yield and composition. Although FGF2 stimulation enhances the enrichment of neuroprotective EV cargo, the optimal concentration and duration of stimulation may require empirical optimization for different experimental contexts. Furthermore, the characterization methods employed—TEM, NTA, and Western blotting—confirm vesicle identity and quality but do not definitively identify the biogenesis pathways of EVs to enable further subclassification. While this protocol is established for astrocytes, its applicability to other cell types may necessitate adjustments in culture conditions and EV isolation parameters. Finally, all procedures should be performed under sterile conditions, and researchers should be mindful of potential batch-to-batch variability when scaling up for large-scale preparations.

## Troubleshooting

### Problem 1

Low cell yield or poor viability after plating (steps 1–14).

### Potential solution

• Ensure complete removal of meninges during dissection, as residual meninges can lead to fibroblast overgrowth and reduced astrocyte purity.
• Confirm that dissection is performed on ice and that tissue is kept cold throughout the procedure to preserve cell viability.
• Optimize trypsin digestion time and temperature; over-digestion damages cells, while under-digestion results in insufficient cell release.

### Problem 2

Inconsistent astrocyte purity across batches (steps 1–14).

### Potential solution

• Use neonatal mice (P0-P3) for consistent cell yield and purity.
• Passage cells at least twice before experiments to stabilize the astrocyte phenotype; use cells within five passages to avoid phenotypic drift.

### Problem 3

Low EV yield after FGF2 stimulation (steps 15–18).

### Potential solution

• Optimize FGF2 concentration (e.g., 2–20 ng/mL) and stimulation duration, as these parameters may affect EV secretion.
• Ensure the use of extracellular vesicle-depleted serum during EV collection to avoid contamination with serum-derived vesicles.
• Collect conditioned medium when cells are at 70–80% confluence; over-confluent cultures exhibit altered secretion profiles.

### Problem 4

Inconsistent NTA results (size distribution or concentration) (steps 36–37).

### Potential solution

• Dilute the EV sample appropriately to achieve an optimal particle concentration (typically 10^5^–10^9^ particles/mL) for accurate tracking.
• Use gentle pipetting to resuspend the sample; avoid vortex, which can cause aggregation and alter size measurements.
• Maintain consistent temperature (e.g., 25°C) during measurement, as temperature fluctuations affect particle motion and calculated size.

### Problem 5

Weak or non-specific signals in Western blotting (steps 38–45).

### Potential solution

• Confirm sufficient EV protein is loaded; low yields require concentration of the sample before lysis.
• Include positive controls (e.g., cell lysates expressing EV markers) to validate antibody performance.
• Ensure proper membrane blocking and washing to reduce background; use 5% skim milk in TBST for blocking.

## Resource availability

### Lead contact

Further information and requests for resources and reagents should be directed to and will be fulfilled by the lead contact, Ye Xi (ye.xi@fmmu.edu.cn).

### Technical contact

Operational inquiries regarding protocol implementation should be addressed by the technical contacts, Xiaomin Wen (wenxmnwu@163.com) and Ye Xi (ye.xi@fmmu.edu.cn).

### Materials availability

This study did not produce novel reagents.

### Data and code availability

This study did not generate datasets/code.

## Acknowledgments

This study was supported by the 10.13039/100014717National Natural Science Foundation of China (82571429 to Y.X. and 82221001 to S.W.), the Natural Science Basic Research Program of Shaanxi Province (2024JC-YBQN-0774 to Y.X.), and the Open Fund of Key Laboratory of Clinical Neurology, Ministry of Education (2025A02 to Y.X.). Select objects were adapted from BioGDP in the graphical abstract.

## Author contributions

Y.X. and S.W. conceived the project and acquired funding to support this work. X.W., Y.X., and M.W. conducted the experiments and wrote the manuscript. M.W. and W.C. completed part of the experiments. M.W. and Z.G. completed the drawing of the graphical abstract.

## Declaration of interests

The authors declare no competing interests.
